# Coronavirus-linked pregnancy complications: a comparative study

**DOI:** 10.1186/s43042-022-00229-5

**Published:** 2022-01-30

**Authors:** Miapeh Kous Gonlepa, Olayemi Hafeez Rufai, Chidinmma Grace Ofuonye, Lapologang Sebaka

**Affiliations:** 1 Department of Public Administration, School of Public Affairs, University of Science and Technology of China, Hefei, China; 2 Department of Philosophy of Science and Technology, School of Public Affairs, University of Science and Technology of China, Hefei, China

**To the Editor**,

## Main text

During the outbreak of infectious diseases, pregnant women and their fetus are among the high risk population. Currently, disease outcomes for neonates and pregnant women with COVID-19 infection have been reported in the literature, with no direct evidence of vertical transmission [1]. Mechanical and physiological changes in the state of pregnancy generally increases the rate of susceptibility to infections, especially when the cardiorespiratory system is affected. This encourages a quick progression to respiratory failure in the gravida [1]. In addition, the bias in pregnancy towards the system dominance of the T-helper 2 (Th2) increases the vulnerability of the mother to viral infections, as the T-helper 2 system protects the fetus. These specific challenges mandate an integrated approach to pregnancies affected by the SARS-CoV-2 [1, 2]. Here, we highlight different reported pregnancy complications as a result of infection by the three known human coronaviruses, while emphasizing on the need to prevent worse complications in the current pandemic. This necessitates the provision of essential health care for pregnant women amid the current COVID-19 pandemic.

Severe Acute Respiratory Syndrome (SARS) is a disease caused by the Severe Acute Respiratory Syndrome Coronavirus (SARS-CoV). The emergence of the SARS-CoV was first reported in February 2003, with the first set of observed cases in the Guangdong Province of China [3]. Almost 30 countries around the world were affected, leading to over 8000 reported infection cases and 770 deaths [3]. Hong Kong recorded the largest case series of SARS-CoV-infected pregnant women, where 12 pregnant women were detected to be infected, resulting into 3 deaths [4]. Laboratory and clinical observations were similar to observations in the non-pregnant population. Computed tomography or pneumonia on chest radiograph was observed in patients, and other observed medical complications included sepsis in 2 patients, secondary bacterial pneumonia in another 2, renal failure in 3 patients, disseminated intravascular coagulopathy (DIC) in another 3, while the remaining 4 patients experienced adult respiratory distress syndrome [4].

Middle East Respiratory Syndrome (MERS) is a respiratory illness caused by the Middle East Respiratory Syndrome Coronavirus (MERS-CoV). MERS was identified first in 2012 in Saudi Arabia, where it spread to other Arabian Peninsula countries and beyond; including the United States [5]. Outside the Arabian Peninsula, the Republic of Korea experienced the largest outbreak of the disease in 2015 [5]. Although, there are limited information on the MERS infection among pregnant women, Rasmussen et al. [3] however identified 13 reported cases of MERS-CoV-infected pregnant woman from different countries. In Saudi Arabia, 8 patients where identified, while 2 were identified in Korea, 1 in Jordan, 1 in the United Arab Emirates and 1 in the Philippines [3]. Two women out of the 13 reported cases were asymptomatic, while the manifestations from the remaining 11 patients that showed symptoms were observed to be similar to the observed manifestations in the nonpregnant MERS-CoV-infected patients. Seven out of the 13 infected patients were admitted either for acute respiratory distress syndrome or respiratory deterioration at an intensive care unit while 5 needed ventilator support. Three deaths were eventually recorded while 8 of the patients recovered fully [3]. Among the 3 recorded deaths, the mothers died between 8 and 25 days after delivery. The babies born to the two asymptomatic mothers were born healthy at term. As for the mothers that showed symptoms, one intrauterine fetal demise was recorded alongside one stillbirth. A baby was delivered at 25 weeks but eventually died after 4 h. Two healthy preterm infants and 5 healthy term infants were also recorded [3].

The COVID-19 pneumonia pandemic is caused by the Severe Acute Respiratory Syndrome Coronavirus 2 (SARS-CoV-2) and it is spreading globally at a rapid rate, with a basic R0 (reproduction number) ranging between 2 and 2.5. This implies that 2–3 people have high chances of being infected from an index patient [6]. The socioeconomic and morbidity impact of the disease made it necessary for drastic measures to implemented across all continents of the world, such as boarder closures and nationwide lockdown [6]. Compared to the SARS and MERS, the outcome of the COVID-19 pandemic on pregnant women appear more promising. According to pooled data, the impact of COVID-19 on pregnant women revealed a case fatality rate of 0%, compared to SARS and MERS, with case fatality rates of 18% and 25% respectively [7]. Compared to uninfected pregnant women, symptomatic pregnant women with COVID-19 that also required hospitalization displayed worse maternal outcomes, with a higher risk of death, although the death risk remains low (maternal mortality rate from COVID-19 in the UK is 2.2 per 100,000 maternities) [8]. In the SARS and MERS diseases, severe sepsis and progressive respiratory failure were the most common causes of recorded deaths. This is not surprising, considering the predisposition to superimposed bacterial infections due to respiratory microbiome alterations after viral pneumonia, immune response dysregulation and direct mucosal injuries [9]. Fetal complications resulting from the SARS-CoV-2 infection of pregnant women include miscarriage (2%), intrauterine growth restriction (10%), and preterm birth (39%). Fever with a median temperature between 38.1 and 39.0 °C is also a common symptom associated with the COVID-19 [1]. In a study by Pierce-Williams et al. [10] which specifically reported outcome by the severity of the disease, 32 out of the 64 hospitalized pregnant women for critical or severe COVID-19 delivered in the disease state. 13 deliveries among the 20 pregnant women with critical disease and 9 deliveries among the 44 pregnant women with severe disease were as a result of maternal status while only 3 were delivered as a result of fetal status [10]. Preterm birth was experienced in 75% of the pregnant women with critical disease while only 9% of the pregnant women with severe disease experienced preterm birth. A similar study by Metz et al. [11] also revealed a higher risk of preterm and cesarean birth in critical and severe disease states.

## Conclusion

Pregnant women are uniquely vulnerable in any outbreak of infectious diseases due to their changed physiology, compromised immunological and mechanical functions, and susceptibility to infections. The need to protect the fetus also increases the challenges of managing their health. Although, from current reports, it is difficult to make absolute conclusions on the possibility pregnant women being at high risk of the severe consequences of COVID-19. However, special precautions are still required for the minimization of infection in pregnant women and for the prevention of possible vertical transmission to their fetus.

## Data Availability

Not applicable.

## Abbreviations

SARS: Severe Acute Respiratory Syndrome
MERS: Middle East Respiratory Syndrome
SARS-CoV: Severe Acute Respiratory Syndrome Coronavirus
MERS-CoV: Middle East Respiratory Syndrome Coronavirus
SARS-CoV-2: Severe Acute Respiratory Syndrome Coronavirus 2
COVID-19: Coronavirus Disease 19
DIC: Disseminated Intravascular Coagulopathy
R0: Reproduction number
